# Pleasurable music affects reinforcement learning according to the listener

**DOI:** 10.3389/fpsyg.2013.00541

**Published:** 2013-08-21

**Authors:** Benjamin P. Gold, Michael J. Frank, Brigitte Bogert, Elvira Brattico

**Affiliations:** ^1^Cognitive Brain Research Unit, Institute of Behavioral Sciences, University of HelsinkiHelsinki, Finland; ^2^Department of Music, Finnish Center of Excellence in Interdisciplinary Music Research, University of JyväskyläJyväskylä, Finland; ^3^Department of Cognitive, Linguistic and Psychological Sciences, Brown UniversityProvidence, RI, USA; ^4^Brain and Mind Laboratory, Department of Biomedical Engineering and Computational Science, Aalto UniversityEspoo, Finland

**Keywords:** music, pleasure, reinforcement learning, reward, dopamine, subjectivity, musical experience, listening strategy

## Abstract

Mounting evidence links the enjoyment of music to brain areas implicated in emotion and the dopaminergic reward system. In particular, dopamine release in the ventral striatum seems to play a major role in the rewarding aspect of music listening. Striatal dopamine also influences reinforcement learning, such that subjects with greater dopamine efficacy learn better to approach rewards while those with lesser dopamine efficacy learn better to avoid punishments. In this study, we explored the practical implications of musical pleasure through its ability to facilitate reinforcement learning via non-pharmacological dopamine elicitation. Subjects from a wide variety of musical backgrounds chose a pleasurable and a neutral piece of music from an experimenter-compiled database, and then listened to one or both of these pieces (according to pseudo-random group assignment) as they performed a reinforcement learning task dependent on dopamine transmission. We assessed musical backgrounds as well as typical listening patterns with the new Helsinki Inventory of Music and Affective Behaviors (HIMAB), and separately investigated behavior for the training and test phases of the learning task. Subjects with more musical experience trained better with neutral music and tested better with pleasurable music, while those with less musical experience exhibited the opposite effect. HIMAB results regarding listening behaviors and subjective music ratings indicate that these effects arose from different listening styles: namely, more affective listening in non-musicians and more analytical listening in musicians. In conclusion, musical pleasure was able to influence task performance, and the shape of this effect depended on group and individual factors. These findings have implications in affective neuroscience, neuroaesthetics, learning, and music therapy.

## Introduction

### From musical pleasure to reinforcement learning

The emotional power of music is evident from music downloads to band T-shirts, from film scores to music therapy, and from concert sales to any Friday or Saturday night out. Despite having no intrinsic biological or tangible value, music is profoundly important to people of all cultures and all walks of life (Sloboda and Juslin, 2001); listening to music is consistently ranked as one of the most rewarding human experiences (Dubé and Le Bel, 2003). Influential theories of emotion describe pleasure as an integral part of core affect (Lindquist et al., 2012) or survival functions (LeDoux, 2012), and neuroimaging evidence links pleasurable music listening with brain areas implicated in emotion and the dopaminergic reward system (Blood et al., 1999; Blood and Zatorre, 2001; Menon and Levitin, 2005; Salimpoor et al., 2011). Accordingly, people primarily listen to music for emotion and mood regulation (Sloboda and O'Neill, 2001; Saarikallio and Erkkilä, 2007), suggesting a possible functional role of musical pleasure. Nonetheless, with implications in affective neuroscience, neuroaesthetics, and music therapy, the practical ramifications of musical pleasure remain unclear.

Can music direct reward-based decision making? Although the famous “Mozart effect” implies that music can temporarily influence cognitive performance (Rauscher et al., 1993), its functional relationship to reward processing has not yet been assessed. How do different people experience pleasure, and does it affect them differently? Musical emotions are highly subjective and preferences for certain musical pieces or genres vary widely across individuals (Rentfrow and Gosling, 2003; Eerola and Vuoskoski, 2011), yet these differences are often treated as random noise or emergent states (cf. Brown et al., 2011; Kühn and Gallinat, 2012). We explored the reward implications of subjective musical pleasure through its ability to affect reward-based learning.

**Figure 1:**
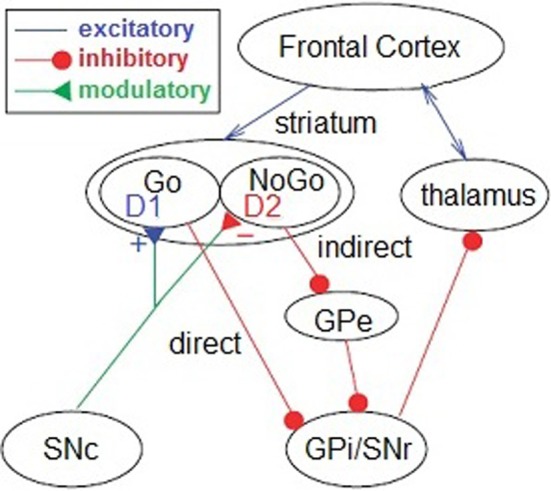
**Reinforcement learning model.** In the reinforcement learning model (from Frank et al., 2004), phasic increases in dopamine promote action selection in the thalamus via the D1-receptor “Go” pathway, whereas phasic decreases promote action avoidance via the D2-receptor “NoGo” pathway. Both processes originate in the striatum and receive cortical and subcortical inputs. SNc, substantia nigra pars compacta; GPi, internal segment of the globus pallidus; GPe, external segment of the globus pallidus; SNr, substantia nigra pars reticula.

Reinforcement learning is driven by dopaminergic reward prediction errors that signal the discrepancy between expected and experienced action outcomes (Montague et al., 1996; Schultz, 2002). Learning occurs as behavioral modifications reflect and ultimately minimize these prediction errors over time (Hollerman and Schultz, 1998). In one model, the selection of rewarded actions is promoted by Hebbian potentiation of a direct D1-receptor “Go” pathway following phasic increases in dopamine, while action avoidance is achieved via potentiation of an indirect D2-receptor “NoGo” pathway following phasic decreases (Figure 1; Frank et al., 2004). Genetic, pharmacological, and neuropsychiatric research converge to show that learning and decision making are preferentially guided by rewards in subjects with greater striatal dopamine efficacy and preferentially guided by punishments in those with lesser dopamine efficacy (Frank et al., 2004, 2007a; Jocham et al., 2011; Shiner et al., 2012).

### The neural bases of reinforcement learning and musical pleasure

The neural bases of reinforcement learning center around the striatum (especially the nucleus accumbens; NAc) and the ventromedial prefrontal cortex (vmPFC). Combining functional magnetic resonance imaging (fMRI) and positron emission tomography (PET), Schott and colleagues (2008) found that reward anticipation corresponded to dopaminergic activity in the substantia nigra and the ventral tegmental area, whereas reward itself elicited dopamine release in the ventral striatum and especially the NAc. The magnitudes of the anticipatory and reward-related dopamine release were correlated, as the NAc is the target of dense projections from the ventral tegmental area. Many studies have also shown with fMRI that striatal and ventral tegmental learning activity reflect reward prediction errors (O'Doherty et al., 2004; Daw and Doya, 2006; D'Ardenne et al., 2008; Caplin et al., 2010; Badre and Frank, 2012). Moreover, such prediction error activity is modulated by dopaminergic drug administration and predictive of behavioral measures of learning (Jocham et al., 2011). This latter study also revealed vmPFC activity associated with both rewards and the learned values of rewarded stimuli, implicating this area in the tracking of learned reward values over time. Similarly, research with dopamine deficiency in Parkinson's disease showed that NAc and vmPFC activity during reinforcement learning correlated with the value of the chosen stimulus, but only in patients who had taken dopaminergic medications (Shiner et al., 2012).

Unsurprisingly, these reward areas correspond to those active in pleasurable music listening. Blood and colleagues (1999) first linked pleasant music to increased limbic activity in areas including the orbitofrontal cortex and vmPFC, and a subsequent investigation demonstrated that intensely pleasurable responses to music correlated with increased activity in the ventral striatum and thalamus and decreased activity in the amygdala, hippocampus, and vmPFC (Blood and Zatorre, 2001). Salimpoor and colleagues (2011) further showed with PET and fMRI that pleasurable music listening activated the striatum, and that peak experiences of pleasure increased dopamine release in the NAc. Crucially, the authors confirmed that this activity represented dopamine transmission by demonstrating that peak pleasure epochs corresponded to peak NAc dopamine release. They also showed that increases in subjective pleasure were correlated with NAc activity even in the absence of the stereotypical “chills” responses they used to index intense pleasure, suggesting that this relationship extended beyond only peak pleasure experiences.

Dopamine is not directly related to hedonic experience, however, as discussed above it is related to positive deviations from expectation rather than reward *per se* (for reviews see Schultz, 2002; Berridge and Kringelbach, 2013). Notably, music generally evokes emotions through the manipulation of cognitive expectations, and pleasurable music is often pleasurable because of how it builds, meets, and defies these expectations (Meyer, 1956; Huron, 2006; Vuust and Kringelbach, 2010). Indeed, “chills” strongly correlate with moments of expectancy violation (Sloboda, 1991). These expectations can come from top–down explicit knowledge of a musical piece or from bottom-up implicit schematic predictions based on previous experiences within a musical genre or schema (Bharucha, 1994; Huron, 2006), which can account for some subjectivity of musical preferences and enjoyment of both familiar and unfamiliar music. Thus, the activity of the NAc during pleasurable music listening can be thought of as reward prediction errors, with pleasant musical surprises reflecting large positive errors. Consistent with this interpretation, an effective connectivity analysis of subjects listening to pleasant as opposed to scrambled musical excerpts revealed significant interactions between the NAc and the right middle temporal and superior temporal gyri (Menon and Levitin, 2005), which are involved in the perception of schematic tonal structures (Zatorre et al., 1994). Moreover, a recent investigation showed that the subjective reward value of different musical pieces could be predicted by increased functional connectivity between the NAc and brain regions involved in auditory schematic processing, valuation, and emotional processing, suggesting that music enjoyment depends on previously stored acoustic information and the positive prediction errors that arise from these preconceptions (Salimpoor et al., 2013). While reward prediction errors are probably not the only cause of dopamine release during pleasurable music listening and more research is needed to substantiate this “predictive coding model” of aesthetic enjoyment (Van de Cruys and Wagemans, 2011), the aforementioned studies suggest that the overlapping activation patterns of musical pleasure and reward-based learning may thus reflect a common reliance on reward prediction errors.

### The origins of the idiosyncratic nature of musical pleasure

Musical expectations differ greatly from genre to genre and person to person, and musical preferences vary even more. Personality traits, intelligence, and various social factors can all influence musical tastes (Rentfrow and Gosling, 2003; Chamorro-Premuzic and Furnham, 2007; Chamorro-Premuzic et al., 2012). During listening, online differences in the perception of music or in music-directed attention could also affect musical preferences (Kantor-Martynuska and Fajkowska, in preparation).

Of the many interpersonal influences on musical preferences, past musical experience has received the most attention, with both informal musical activities and formal training corresponding to variations in perceptual, cognitive, and affective responses to music (e.g., Tervaniemi et al., 2006; Chapin et al., 2010; Dellacherie et al., 2011; Brattico and Pearce, 2012; Oechslin et al., 2012; Seger et al., 2013). Musical experience also affects structural development, functional connectivity, and listening strategies at the neural level (Gaser and Schlaug, 2003; Koeneke et al., 2004; Bengtsson et al., 2005; Chen et al., 2008; Hyde et al., 2009; for reviews see Rodrigues et al., 2010; Levitin, 2012). For example, music experts tend to describe musical aesthetics with music-specific adjectives (such as melodic, rhythmic, and harmonic) whereas non-musicians rely more on emotion-related adjectives (Istók et al., 2009). Electrophysiological evidence also suggests that music experts utilize more analytical strategies than non-musicians when giving aesthetic judgments of chord sequences, while the latter instead respond more emotionally (Müller et al., 2010). Yet while musical expertise is associated with greater engagement in music as a primary focus, musicians are not necessarily more likely to be distracted by music (Kantor-Martynuska and Fajkowska, in preparation). Notably, many of these effects are correlational, meaning that they form a spectrum along musical experience from non-musicians to amateur musicians to musicians (Gaser and Schlaug, 2003; Tervaniemi et al., 2006; Hyde et al., 2009; Oechslin et al., 2012).

### Study aims and hypotheses

In the present study, we first aimed to assess the practical implications of musically elicited dopamine by determining whether musical pleasure could facilitate reinforcement learning via non-pharmacological dopamine elicitation. To this end, we played pleasurable and neutral music for participants during a reinforcement learning task dependent on dopamine transmission (Frank et al., 2004). Given the emotional power of music and its capacity to activate the mesocorticolimbic reward system, we expected musical pleasure to influence reinforcement learning by evoking a dopaminergic response that would enhance appetitive behaviors.

We also expected music's influence to depend on the musical background and listening patterns of the individual. We combined pre-existing and novel self-report measures to objectively identify individual musical experiences with a new Helsinki Inventory of Music and Affective Behaviors (HIMAB). With this, we sought to explore the relationships between subjective musical pleasure, diverse musical backgrounds, and music listening patterns. During the learning paradigm, we hypothesized that musically inexperienced subjects would be more emotionally affected by the music they enjoyed and thus benefit more from listening to it during the task, whereas more musically experienced subjects would think about the music more analytically during learning and thus divert focus from the learning task.

## Materials and methods

### Subjects

This experiment was approved by the local ethics committee of the University of Helsinki. Ninety volunteers (33 males, mean age = 27.5 ± 6.0 years) participated. They had no hearing or neurological disorders, spoke and read English fluently, gave informed consent, and received “culture passes” of monetary value in compensation for their time. Seventeen of these volunteers (18.9%) failed to perform significantly above chance by the end of training, and so the data described hereafter pertain to the remaining 73 (26 males, mean age = 27.1 ± 5.8 years).

We grouped subjects according to the music they would hear during the training and test phases of the reinforcement learning task. This process was pseudo-random in order to ensure that each group had similar distributions of musical experience (Table 1). The “NP” group listened to neutral music as they learned and pleasurable music as they generalized their knowledge to the test. The opposite group, “PN,” listened to pleasurable music during training and neutral music during the test. To control for learning degradation due to state dependencies (Overton, 1966), we included two groups that listened to the same music for both training and testing (“NN” and “PP”). The presence of music in general likely distracted participants and worsened overall task performance, but this experiment specifically concerned the comparison of different emotional responses to music. As such, we examined only within-music effects.

**Table 1:** **Experimental groups**.

**Group**	**Subjects**	**Mean playing years ± *SD***	**Playing years range**	**Mean weekly listening ± *SD* (h)**	**Weekly listening range (h)**	**Mean age ± *SD* (years)**
NN	19 (7 male)	7.3 ± 6.9	0–19	19.6 ± 15.8	2.5–70	27.6 ± 6.0
NP	19 (8 male)	9.1 ± 8.2	0–26	18.4 ± 17.8	2.5–70	27.8 ± 6.7
PN	18 (5 male)	9.0 ± 7.6	0–24	19.1 ± 24.9	2–110	26.0 ± 3.2
PP	17 (5 male)	12.2 ± 9.8	2–39	14.3 ± 15.9	2 – 49	26.7 ± 6.7
Total	73 (26 male)	9.3 ± 8.2	0–39	17.9 ± 18.7	2–110	27.1 ± 5.8

To simplify our sample and investigate musicianship more closely, we also classified subjects according to their musical backgrounds. We defined musicians as participants who had earned a music degree and/or received compensation for performing music with at least 5 years of recent (within the last 5 years) and weekly playing or singing experience; this experiment included 23 such musicians. Amateur musicians had between 1 and 5 years of recent and weekly musical experience or more than 5 years of experience (potentially including a music degree) that had not been recent and/or at least weekly; there were 22 amateur musicians in this study. Non-musicians had fewer than 5 years of musical experience that was not recent and/or weekly; we analyzed data from 28 non-musicians. Table 2 provides more information about the musical backgrounds in this experiment.

**Table 2:** **Musical backgrounds**.

**Classification**		**Playing years**	**Years ago**	**Pro/student years**	**Years ago**
Musicians	Mean ± SD	16.8 ± 6.9	2.7 ± 5.5	8.9 ± 4.8	2.2 ± 5.2
Amateurs	Mean ± SD	11.4 ± 5.0	6.7 ± 8.1	1.9 ± 3.2	7.6 ± 5.2
Non-musicians	Mean ± SD	1.6 ± 2.2	10.4 ± 6.2	0.0 ± 0.0	N/A

### Listening test

Prior to the experimental task, each subject was required to complete a listening test at home. This test involved listening to and rating 14 songs from an experimenter-created list of instrumental film score pieces (Table A1 in Appendix) that were sent to the subjects via online file sharing upon their consent to participate in the experiment. The musical pieces came from a database previously rated by 116 listeners (Eerola and Vuoskoski, 2011) and were chosen for this experiment because of similar valence (mean rating = 5.56 out of 9 ± 0.80), energy (mean rating = 2.61 out of 9 ± 0.61), and tension (mean rating = 2.33 out of 9 ± 0.81) ratings by those listeners. Subjects in the present study evaluated the familiarity, pleasantness, and arousal of each piece on five-point Likert scales as they listened, repeating each piece until they were satisfied with their ratings. They then chose their three favorite pieces and three pieces about which they felt completely neutral from the list. Using their ratings (i.e., selecting pieces with similar affective ratings) and excluding any songs they explicitly recognized, we chose one of their favorite pieces to be their pleasurable music and one of their neutral pieces to be their neutral music during the experiment. We also ensured that each piece was used both as pleasurable music and as neutral music; for the 73 subjects, each piece served as pleasurable music an average of 5.21 ± 2.89 times, and as neutral music 5.21 ± 2.49 times. This way, each subject's pleasurable music was another subject's neutral music and vice-versa. We compared the pleasurable music and neutral music ratings with paired-samples *t*-tests and found that, in spite of our attempt to match the affective ratings of the pleasurable and neutral pieces of music, subjects rated their pleasurable music higher in each category (familiarity, pleasantness, and arousal; all *p*s < 0.05; Table 3). In addition, independent samples *t*-tests revealed that non-musicians rated the pleasantness of their pleasurable music higher (mean rating = 4.71 ± 0.46) than musicians did (mean rating = 4.39 ± 0.58; *t*_(49)_ = −2.21, *p* < 0.05). We accounted for these differences by using the listening test ratings as covariates in repeated-measures analyses of covariance (ANCOVAs). This way, any main effects or interactions we observed with respect to musical conditions reflected subjective differences of music enjoyment and not familiarity, pleasantness, or arousal. We also performed multiple linear regression analyses with the ratings as regressors to examine the influence these ratings had on task performance.

**Table 3:** **Listening test results**.

***T*-test**	**Pleasurable music mean rating ± standard deviation**	**Neutral music mean rating ± standard deviation**	**T_(72)_**	***P*-value**
Familiarity	2.30 ± 1.27	1.95 ± 1.09	4.05	*p* = 0.0001
Pleasantness	4.53 ± 0.58	2.96 ± 0.79	15.28	*p* < 0.0001
Arousal	2.95 ± 1.21	2.56 ± 0.88	2.59	*p* < 0.05

### Probabilistic selection paradigm

**Figure 2:**
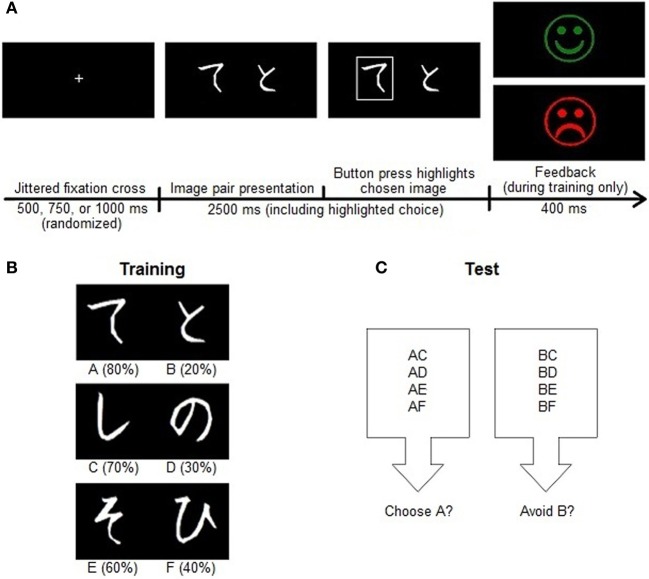
**Probabilistic selection (PS) task. (A)** Each trial in the PS task began with a jittered fixation cross followed by a pair of stimuli for 2500 ms. Following a left or right button response, the selected image appeared highlighted on the screen for the duration of the 2500 ms presentation. Choices during training then received probabilistic feedback, whereas those during testing were followed by the fixation cross marking the next trial. **(B)** In the training phase, participants learned to choose between three discrete pairs of Japanese Hiragana characters with different reward contingencies. Each pair had a better and worse choice, but the relative weights of these values changed. The reward probabilities of each stimulus are shown in parentheses. **(C)** In the test phase, participants generalized their knowledge of the training pairs to recombined stimulus pairs. There was no feedback in this phase. Learning to choose A over B during training could reflect approach learning, avoidance learning, or both, and so we assessed overall test performance as well as the accuracy of **(A)** choices and **(B)** avoidances when these stimuli appeared in novel pairs during testing.

The probabilistic selection (PS) task (Figure 2), adapted from Frank and colleagues (2004), took place at the University of Helsinki. Subjects sat in a soundproof room approximately one meter from a computer monitor while the experiment was delivered using Presentation software (Neurobehavioral Systems, Ltd.). The pre-selected musical pieces played binaurally through headphones at a comfortable intensity, and each piece looped to ensure that music played throughout the entire durations of the training and test phases of the task. The visual stimuli were Japanese Hiragana characters.

This task had two phases. In the training phase, three different image pairs appeared on the screen in random order. Each image had a different probabilistic chance of reward. The pairs (and their reward contingencies) were termed AB (image A had an 80% probability of reward and image B had a 20% probability), CD (70 and 30%), and EF (60 and 40%). The image pair appeared for 2500 ms after a jittered fixation cross of 500, 750, or 1000 ms, and presentations were counterbalanced so that each image occurred just as often on each side of the screen. Although the subjects had no prior information about the stimuli (screening ensured no experience with the Japanese language), they were instructed to choose between them with the left or right button on a button box. If the subject failed to respond within the allotted time window, white text reading “No Response” appeared on the screen. Otherwise, upon the event of a button press, a white rectangle appeared around the selected image for the remainder of the 2500-ms stimulus duration. Subjects then received either “correct” (a green smiley face) or “incorrect” (a red sad face) feedback for 400 ms based on a random draw of the images' inherent reward contingencies.

Before starting the training phase, subjects acclimated themselves to the paradigm, the music volume, and the button box with eight practice trials. These were identical to the training trials, except they used different Hiragana characters and reward certainties (100 or 0%) instead of probabilities. The four images in the practice session each appeared twice, once on each side of the screen, in discrete pairings termed WX and YZ. When the practice session finished, the experimenter ensured that the subject understood the task and that the music intensity was comfortable, and offered to answer any questions about the paradigm. The training phase began when the participant was ready. Training was divided into three blocks of 54 stimulus pairs each with participant-paced rest breaks in between. With this design, subjects encountered each stimulus pair 18 times in each training block.

Learning to choose A over B involves learning that choosing A results in positive feedback (approach or “Go” learning), that choosing B results in negative feedback (avoidance or “NoGo” learning), or both. The test phase of this task thus assessed the extent to which participants had learned about the positive and negative outcomes of their choices and were able to transfer or generalize this knowledge. The stimuli from the training phase were recombined such that all 15 possible pairings occurred during the test. The test consisted of 90 trials without feedback, with all image pairs occurring six times (three times in each order).

### Helsinki inventory of music and affective behaviors (HIMAB)

Subjects completed the (HIMAB; Table A2 in Appendix) either before or after the PS task. Although most subjects did this at home, the time and location of HIMAB administration depended on the subject's availability. Since this inventory reflects previous musical experiences and typical listening patterns, we do not suspect that answers were affected by different response contexts. In addition, the experimenter was available for questions even when the inventory was done at home and before the subjects submitted their responses.

The first component of the HIMAB assesses musical experience with questions regarding the intensity, regularity, duration, and time since any musical training, professional musical experience, or working toward a musical degree. We used these questions to derive the variable “Playing Years,” as a measure of how many years each subject played/has played music (including singing). A question on the frequency of music listening represented the “Weekly Listening Hours” variable, which measured any and all kinds of music listening behavior in a typical week. The rest of the inventory corresponds to continuous variables for covariance and regression analyses. Several of these variables came from three pre-existing scales. The Music Consumption Scale (“music consumption”) quantifies how much live music the subject hears and purchases/downloads on a regular basis (Chamorro-Premuzic et al., 2012). The Uses of Music Inventory (UMI; Chamorro-Premuzic and Furnham, 2007) assesses the extent to which the subject uses music for emotional, cognitive, and social/background purposes (“emotional use of music,” “cognitive use of music,” and “background use of music”). The Music-Directed Attention Scale (MDAS; Kantor-Martynuska and Fajkowska, in preparation) measures the subject's tendency to have music divert attention from tasks of primary focus (“music distractibility”) and the extent of the subject's engagement in music when it is the primary focus (“music engagement”).

“Music importance,” “active listening,” and “passive listening” were novel variables in the HIMAB. For “music importance,” subjects rated on a seven-point Likert scale (from “Not at all important” to “Very important”) how important music is in their daily lives. Whereas people may listen to or consume music to various extents and for various reasons, “music importance” describes how significant music is on a personal and daily basis and distinguishes, for example, someone who just happens to hear their coworkers' music every day from someone who would miss it if it were absent. For “active listening,” we asked subjects to rate on a seven-point Likert scale how often they listen to music without doing anything else. This variable quantifies the amount of time subjects devote to focused music listening regardless of how important or engaging they might find it. Finally, “passive listening” complements “active listening” by quantifying on the same seven-point Likert scale the amount of time that subjects listen to music while engaged in another activity. Responses throughout the inventory were binary choice, written, or five- or seven-point Likert scales with elaboration available for most questions. Taken together, these variables aimed to comprehensively describe the typical music listening practices of our subjects.

### Statistical tests

We analyzed performance in the PS task with repeated-measures ANCOVAs using accuracy and correct-trial reaction times as dependent variables. We defined accuracy as the proportion of trials in which the subject chose the image with the higher probability of reward, and reaction times as the amount of time between the stimulus onset and the subject's first button press. The individual factors from the HIMAB (music importance, music consumption, emotional use of music, cognitive use of music, background use of music, music distractibility, music engagement, active listening, and passive listening) and the subjective ratings from the listening test (the familiarity, pleasantness, and arousal of the pleasurable and neutral music, treated as six separate variables) served as covariates. Musical Condition (pleasurable and neutral) was a between-subjects factor for both phases, and the musical experience variables Playing Years and Weekly Listening Hours were covariates of interest modeled over the whole sample and then individually for each Musical Condition. In this way, we studied musical experience with continuous variables in order to avoid the problematic classification of musicianship according to arbitrary definitions and the limited statistical power of tests conducted on small musicianship groups. Nonetheless, distinguishing subjects according to their musical backgrounds can simplify and clarify the effects of musical experience, and so we used musicianship categories for these purposes only.

For the training phase, we investigated the process of learning by using Training Block (Block 1, Block 2, and Block 3) and Image Pair (AB, CD, and EF) as within-subjects factors. For the test phase, we defined approach learning as the accurate selection of the most rewarded stimulus, A, whenever it was presented as part of a novel pair (AC, AD, AE, and AF, or “Choose A”) and avoidance learning as the accurate selection of stimuli other than the most frequently punished stimulus, B, whenever it was presented as part of a novel pair (BC, BD, BE, and BF, or “Avoid B”). These measures have repeatedly exhibited differential sensitivities to dopaminergic manipulations (Frank et al., 2004, 2007a,b; Jocham et al., 2011), and so we analyzed accuracy and correct-trial reaction times both for the test phase as a whole and for Choose A/Avoid B conditions in particular. We also investigated the effects of switching and keeping musical conditions between the training and test phases. Hence, Test Condition (Choose A and Avoid B) was a within-subjects factor and Group (NP, PN, NN, and PP) was a between-subjects factor for the test. Finally, we performed planned contrasts using least squared difference tests with Tukey corrections for multiple comparisons and *post-hoc* pairwise comparisons on each significant ANCOVA.

Early behavior in the PS task often includes reacting to the last reward or punishment for a certain stimulus pair by explicitly remembering the event and either seeking it again (“win-stay”) or trying to avoid it (“lose-switch”) the next time it appears (Frank et al., 2007a). This process involves storing previous behaviors and their outcomes in working memory while learning about intervening trials with other stimuli. Although this strategy can be helpful at first, it ultimately proves ineffective due to the probabilistic nature of the task. As such, most subjects abandon it early in training (Frank et al., 2007a). Even so, working memory recruitment could account for differences in task performance, and so we analyzed the frequency of “win-stay” and “lose-switch” choices in the first third of the first training block (18 trials), during which each image pair appeared approximately three times in each order. For this ANCOVA, win-stay/lose-switch frequency was the dependent variable and Musical Condition (pleasurable and neutral) was a between-subjects factor while Playing Years and Weekly Listening Hours were covariates of interest. We also measured baseline performance levels during these trials with ANCOVAs for which accuracy and reaction times were dependent variables and the aforementioned HIMAB variables (music importance, music consumption, emotional use of music, cognitive use of music, background use of music, music distractibility, music engagement, active listening, and passive listening) and the subjective ratings from the listening test (the familiarity, pleasantness, and arousal of the pleasurable and neutral music) were covariates. Playing Years and Weekly Listening Hours were covariates of interest, and we conducted separate *post-hoc* models within each Musical Condition of any significant musical experience-mediated Musical Condition effects. We further explored the relationships between individual musical experiences and PS task performance with multiple linear regression analyses on accuracy and correct-trial reaction times in the training and test phases. The HIMAB variables and listening test ratings served as regressors.

## Results

### General learning in the probabilistic selection paradigm

**Figure 3:**
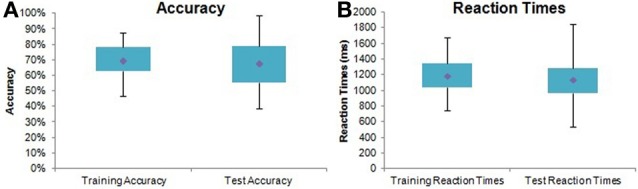
**Probabilistic selection task performance summary.** Box plots with quartiles (upper values 75%, medians 50%, and lower values 25%). The whiskers show the range of the data, with no outliers. **(A)** Overall accuracy in training and testing for all subjects. **(B)** Overall reaction times in training and testing for all subjects.

Seventy-three subjects learned the task significantly above chance as demonstrated by their performance in the third training block [mean accuracy = 77.25 ± 11.81%, single sample *t*-test *t*_(72)_ = 5.24, *p* < 0.0001]. Significant main effects of Training Block [*F*_(2,88)_ = 40.55, *p* < 0.0001] and Image Pair [*F*_(2, 142)_ = 5.25, *p* < 0.01] confirmed that subjects were more accurate in later blocks and with easier (e.g., 80%/20% vs. 60%/40%) pairs. Learning was also evident from reaction times, with subjects responding significantly faster in later training blocks [*F*_(2, 88)_ = 57.73, *p* < 0.0001] and with easier pairs [*F*_(2, 142)_ = 11.69, *p* < 0.0001]. Figure 3 illustrates overall performance on the PS task.

In the test phase, a pairwise comparison (adjusted *p* < 0.05) on a main effect of Test Condition [*F*_(1, 69)_ = 5.94, *p* < 0.05] showed that subjects were significantly more accurate at avoiding B (mean = 68.46 ± 21.06%) than choosing A (mean = 60.02 ± 23.72%). All other test effects varied according to Playing Years, Weekly Listening Hours, Musical Condition, and/or Group, and are thus reported below.

### Effects of musical pleasure on reinforcement learning

The music that subjects listened to during the training phase of the PS task did not significantly affect working memory recruitment as measured by win-stay/lose-switch behavior at the beginning of the phase (*p* > 0.69). Nonetheless, the musical manipulation shaped training performance considerably. Musical Condition did not have an immediate effect on accuracy at the beginning of the training phase (*p* > 0.65), but it did influence accuracy throughout training as a whole [*F*_(1, 18)_ = 7.71, *p* = 0.01]. This result suggests that subjects were more accurate when listening to pleasurable music (mean = 70.24 ± 25.75%) than neutral music (mean = 69.94 ± 25.07%), but a planned comparison of this effect was not significant. Response rates, alternatively, varied according to the music heard during even the beginning of training, with a significant main effect of Musical Condition on initial training reaction times [*F*_(1, 18)_ = 8.20, *p* = 0.01]. A planned comparison for this effect also failed to reach significance. However, a significant main effect of Musical Condition on reaction times throughout training [*F*_(1, 18)_ = 19.53, *p* < 0.0005] showed that subjects listening to the music they rated as pleasurable responded faster (mean = 1158 ± 340 ms) than those listening to the music they rated as neutral (mean = 1198 ± 333 ms; Tukey-Kramer adjusted *p* = 0.01).

**Figure 4:**
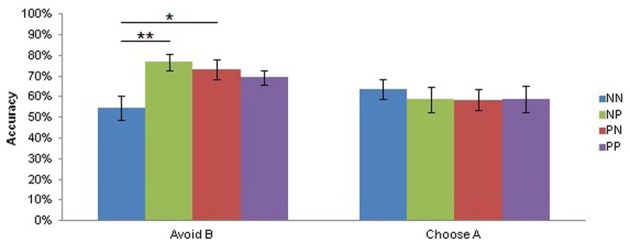
**Test Condition by Group interaction on test accuracy.** There was a significant Test Condition by Group interaction (*p* < 0.05). Subjects did not differ in approach (Choose A) accuracy during the test, but subjects who listened to neutral music during both training and testing (NN) avoided B less accurately than those who listened to neutral music during training and pleasurable music during testing (NP; adjusted *p* < 0.005) and those who listened to pleasurable music during training and neutral music during testing (NP; adjusted *p* < 0.05). Bars depict the mean accuracy for each Group in Choose A and Avoid B conditions, plus or minus the standard error of the mean. PP, subjects who listened to pleasurable music during both training and testing. ^*^p < 0.05; ^**^p < 0.005.

There was also a trend main effect of Musical Condition on test reaction times [*F*_(1, 21)_ = 3.43, *p* = 0.08] suggesting that subjects also responded faster during the test when they listened to pleasurable music (mean = 1149 ± 249 ms) compared to neutral music (mean = 1195 ± 274 ms), but a planned contrast of this effect was not significant. Planned comparisons on a significant Test Condition by Group interaction on Choose A/Avoid B accuracy [*F*_(3, 69)_ = 3.09, *p* < 0.05; Figure 4] showed that the groups were equally adept at Choosing A (NN mean = 63.73 ± 21.07%, NP mean = 58.77 ± 26.86%, PN mean = 58.56 ± 22.02%, PP mean = 58.82 ± 26.14%, all adjusted *p*s > 0.99), but differed in Avoid B accuracy (NN mean = 54.68 ± 25.20%, NP mean = 76.75 ± 17.25%, PN mean = 73.38 ± 19.91%, PP mean = 69.36 ± 13.81%). Specifically, pairwise comparisons of Avoid B accuracy revealed that the NN group performed significantly worse than both the NP (adjusted *p* < 0.005) and the PN (adjusted *p* < 0.05) groups in tests of avoidance learning.

### Effects of musical backgrounds on music-mediated reinforcement learning

**Figure 5:**
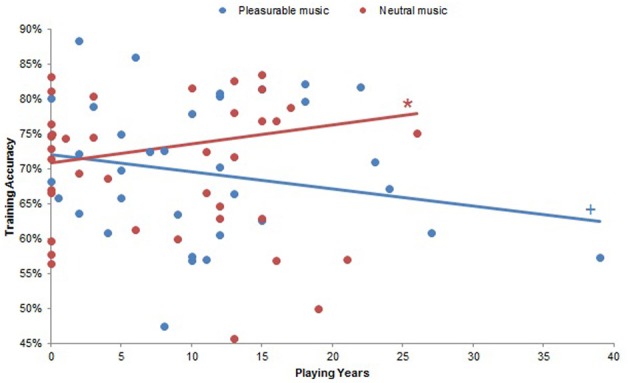
**Playing Years by Musical Condition interaction on training accuracy.** There was a significant Playing Years by Musical Condition interaction on training accuracy (*p* < 0.001). Subjects with more years of musical experience were significantly more accurate when they listened to neutral music (*p* < 0.05), and there was a trend effect of more musically experienced subjects performing less accurately with pleasurable music (*p* = 0.07). ^+^*p* < 0.10; ^*^p < 0.05.

Repeated-measures ANCOVAs with planned comparisons demonstrated several instances in which musical experience modulated the effects of musical pleasure on reinforcement learning. Although musical experiences did not significantly affect accuracy in the beginning of training (all *p*s > 0.17), there was a significant interaction between Playing Years and Musical Condition on accuracy throughout training [*F*_(10, 18)_ = 5.91, *p* < 0.001]. Looking at pleasurable and neutral music separately, we found that accuracy correlated negatively with Playing Years at a trend level for pleasurable music (Beta = −0.08, *p* = 0.07) and significantly and positively with Playing Years for neutral music (β = 0.08, *p* < 0.05). As such, subjects with more musical experience were generally less accurate when they listened to pleasurable music and more accurate when they listened to neutral music (Figure 5). A significant interaction between Weekly Listening Hours and Musical Condition on training accuracy [*F*_(12, 10)_ = 4.03, *p* < 0.05] did not have significant correlations within the separate Musical Conditions (all *p*s > 0.12).

**Figure 6:**
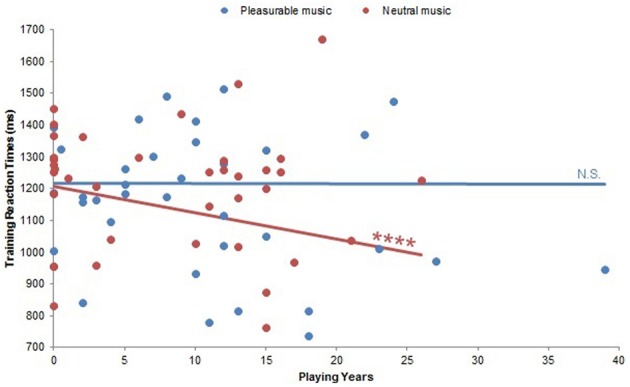
**Playing Years by Musical Condition interaction on training reaction times.** There was a significant Playing Years by Musical Condition interaction on training reaction times (*p* < 0.0001). Within neutral music listening, more musically experienced subjects exhibited faster reaction times (*p* < 0.0001). There was no significant correlation within pleasurable music listening (*p* > 0.95). N.S., not significant; ^****^: *p* < 0.0001.

The effects of Musical Condition were also modulated by musical experience in terms of reaction times. Already in the first 18 training trials, there was a significant Playing Years by Musical Condition interaction on reaction times [*F*_(10, 18)_ = 6.31, *p* < 0.0005]. Pairwise comparisons on this interaction did not reach significance, but a similar significant interaction between Playing Years and Musical Condition on training reaction times [*F*_(10, 18)_ = 15.92, *p* < 0.0001; Figure 6] revealed that subjects with more musical experience responded faster during neutral music listening (β = −0.19, *p* < 0.0001). There was no significant correlation within pleasurable music listening (*p* > 0.95). *Post-hoc* analyses of a significant interaction between Weekly Listening Hours and Musical Condition on training reaction times [*F*_(12, 10)_ = 15.21, *p* < 0.0001] failed to yield any significant correlations (all *p*s > 0.21).

**Figure 7:**
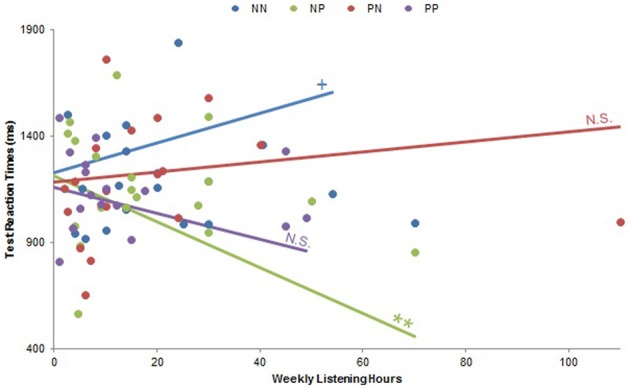
**Weekly Listening Hours by Group interaction on test reaction times.** There was a significant Weekly Listening Hours by Group interaction on test reaction times (*p* < 0.0001). Subjects who listened to music more frequently responded faster when they trained with neutral music and tested with pleasurable music (NP; *p* < 0.01). There was also a trend correlation such that these subjects responded slower when they listened to neutral music during both training and testing (NN, *p* = 0.08). No other within-group correlations were significant (all *p*s > 0.37). PN: subjects who listened to pleasurable music during training and neutral music during testing; PP: subjects who listened to pleasurable music during both training and testing. N.S., not significant; ^+^*p* < 0.10; ^**^p < 0.01.

There were no musical experience by Musical Condition interactions regarding test accuracy (all *p*s > 0.10), but test reaction times exhibited many such effects. A significant Playing Years by Musical Condition interaction [*F*_(7, 21)_ = 3.25, *p* < 0.05] did not yield any significant correlations when we examined pleasurable and neutral music separately, but a related significant Playing Years by Group interaction [*F*_(17, 9)_ = 5.15, *p* < 0.01] examined *post-hoc* within each group demonstrated a significant positive correlation between Playing Years and test reaction times within the NN group (β = 1.26, *p* < 0.001). There were no other significant correlations for this interaction (all *p*s > 0.29), suggesting that both this effect and the aforementioned Playing Years by Musical Condition interaction were driven by more musically experienced subjects responding slower when they listened to neutral music during both training and testing. There was a significant interaction between Weekly Listening Hours and Musical Condition [*F*_(10, 12)_ = 2.83, *p* < 0.05], for which revealed a negative correlation between Weekly Listening Hours and test reaction times within pleasurable music listening was significant at a trend level (β = −0.36, *p* = 0.08), but there was no significant relationship within neutral music listening (*p* > 0.92). Exploration of a significant Weekly Listening Hours by Group interaction [*F*_(20, 16)_ = 10.62, *p* < 0.0001; Figure 7] helped elucidate this effect, exhibiting a significant negative correlation between Weekly Listening Hours and test reaction times within the NP group (β = −0.69, *p* < 0.01), a trend positive correlation within the NN group (β = 0.48, *p* = 0.08), and no other significant relationships (all *p*s > 0.37). Together, these findings indicate that subjects who listen to music more frequently were likely to respond faster if they heard pleasurable music during testing (especially if they had already heard neutral music during training) and slower if they heard neutral music during both training and testing. In other words, more avid music listeners were generally fastest during the test if they were in the NP group, and slowest if they were in the NN group.

As a whole, these reaction time and accuracy effects on training and testing demonstrate that subjects with more music playing and/or listening experience learned better with neutral music but tested better with pleasurable music, with test performance more affected by the music heard during test than that heard during training (NP > PP > PN > NN). From another perspective, subjects with less musical experience performed better when they learned with pleasurable music and tested with neutral music (NN > PN > PP > NP). As such, the NP group was best suited for more musically experienced subjects, and the NN group was best suited for the less musically experienced.

### Helsinki inventory of music and affective behaviors covariates

**Figure 8:**
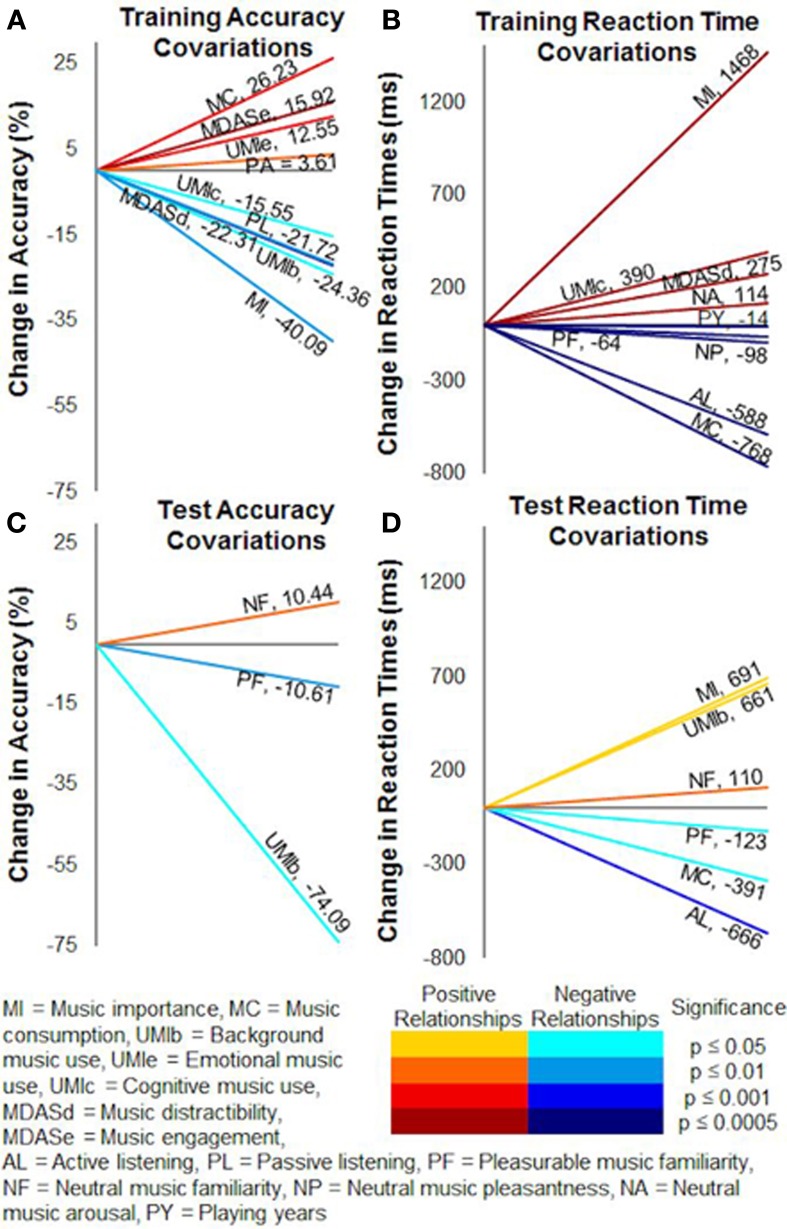
**Covariate relationships on training and test accuracy and reaction times.** Factors from the Helsinki Inventory of Music and Affective Behaviors (HIMAB) and the listening test significantly covaried with probabilistic selection task performance. **(A)** Training accuracy. **(B)** Training reaction times. **(C)** Test accuracy. **(D)** Test reaction times. Numerical values show the slopes of the covariations, and colors represent the directions and significance levels of the effects.

Covariates in the repeated-measures ANCOVAs accounted for individual musical experience and listening differences between the subjects (pooled together) by acting as continuous variables in each analysis. They thus improved the power of the models by removing extraneous influences on variances in accuracy and reaction times. In training, higher accuracies covaried with higher scores of music consumption [*F*_(1, 18)_ = 16.60, *p* < 0.001], emotional use of music [*F*_(1, 18)_ = 3.67, *p* = 0.001], music engagement [*F*_(1, 18)_ = 6.78, *p* < 0.0005], and pleasurable music arousal ratings [*F*_(1, 18)_ = 12.87, *p* < 0.005]. Music importance [*F*_(1, 18)_ = 12.65, *p* < 0.005], background use of music [*F*_(1, 18)_ = 7.16, *p* < 0.05], cognitive use of music [*F*_(1, 18)_ = 5.12, *p* < 0.05], music distractibility [*F*_(1, 18)_ = 22.56, *p* < 0.0005], and passive listening [*F*_(1, 18)_ = 10.83, *p* < 0.005], on the other hand, were negatively related to training accuracy (Figure 8A).

Slower training reaction times (Figure 8B) covaried with higher scores of music importance [*F*_(1, 18)_ = 128.98, *p* < 0.0001], cognitive use of music [*F*_(1, 18)_ = 24.15, *p* = 0.0001], music distractibility [*F*_(1, 18)_ = 26.14, *p* < 0.0001], neutral music familiarity ratings [*F*_(1, 18)_ = 30.50, *p* < 0.0001], and neutral music arousal ratings [*F*_(1, 18)_ = 76.55, *p* < 0.0001]. Training reaction times tended to accelerate as playing years [*F*_(1,10)_ = 42.85, *p* < 0.0001], music consumption [*F*_(1, 18)_ = 108.21, *p* < 0.0001], active listening [*F*_(1, 18)_ = 67.16, *p* < 0.0001], pleasurable music familiarity ratings [*F*_(1, 18)_ = 33.57, *p* < 0.0001], pleasurable music arousal ratings [*F*_(1, 18)_ = 39.77, *p* < 0.0001], and neutral music pleasantness ratings [*F*_(1, 18)_ = 45.28, *p* < 0.0001] increased.

In the test phase, accuracy (Figure 8C) was positively related to neutral music familiarity ratings [*F*_(1, 21)_ = 7.65, *p* = 0.01] and negatively related to background use of music [*F*_(1, 21)_ = 6.73, *p* < 0.05] and pleasurable music familiarity ratings [*F*_(1, 21)_ = 7.26, *p* = 0.01]. Test reaction times (Figure 8D) were generally faster when music consumption [*F*_(1, 21)_ = 4.67, *p* < 0.05], active listening [*F*_(1, 21)_ = 14.98, *p* < 0.001], and pleasurable music pleasantness ratings [*F*_(1, 21)_ = 2.17, *p* < 0.05] increased. Subjects with higher music importance [*F*_(1, 21)_ = 5.62, *p* < 0.05], background use of music [*F*_(1, 21)_ = 4.60, *p* < 0.05], and neutral music familiarity ratings [*F*_(1, 21)_ = 7.36, *p* = 0.01], alternatively, tended to respond slower during the test.

### Helsinki inventory of music and affective behaviors regressors

**Figure 9:**
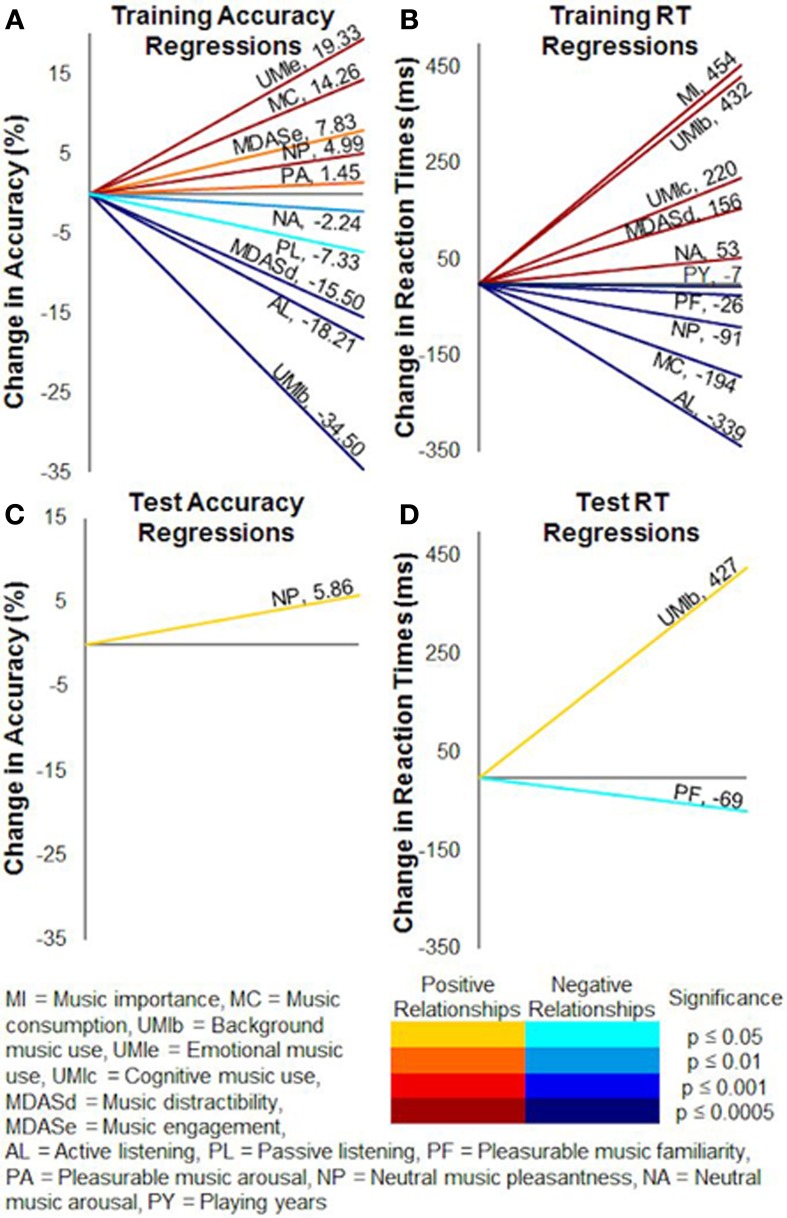
**Multiple regression correlations on training and test accuracy and reaction times.** Multiple linear regressions revealed many individual factors significantly correlated to probabilistic selection task performance. **(A)** Training accuracy. **(B)** Training reaction times. **(C)** Test accuracy. **(D)** Test reaction times. Numerical values show the slopes of the regressions, and colors represent the directions and significance levels of the effects.

We performed multiple linear regression analyses on accuracy and reaction times to further explore the influences of the musical experience and listening variables on task performance. In training, this analysis revealed significant positive correlations between accuracy and music consumption (β = 0.11, *p* < 0.0001), emotional use of music (β = 0.12, *p* < 0.0001), music engagement (β = 0.08, *p* < 0.01), pleasurable music arousal ratings (β = 0.07, *p* = 0.01), and neutral music pleasantness ratings (β = 0.15, *p* < 0.0001). Training accuracy generally decreased when background use of music (β = −0.26, *p* < 0.0001), music distractibility (β = −0.22, *p* < 0.0001), active listening (β = −0.15, *p* < 0.0001), passive listening (β = −0.06, *p* < 0.05), and neutral music arousal ratings (β = −0.08, *p* < 0.01) increased (Figure 9A).

Training reaction times were commonly slower for subjects with higher scores of music importance (β = 0.20, *p* < 0.0001), background use of music (β = 0.24, *p* < 0.0001), cognitive use of music (β = 0.11, *p* = 0.0001), music distractibility (β = 0.17, *p* < 0.0001), and neutral music arousal ratings (β = 0.14, *p* < 0.0001). Subjects with more playing years (β = −0.17, *p* < 0.0001), greater music consumption (β = −0.12, *p* < 0.0001), active listening (β = −0.22, *p* < 0.0001), pleasurable music familiarity ratings (β = −0.10, *p* < 0.05), and neutral music pleasantness ratings (β = −0.21, *p* < 0.0001) tended to respond faster during training (Figure 9B).

Test accuracy was positively correlated with neutral music pleasantness ratings (β = 0.21, *p* < 0.05) (Figure 9C). Slower test reaction times corresponded to higher background use of music scores (β = 0.31, *p* = 0.05) and lower pleasurable music familiarity ratings (β = −0.33, *p* < 0.05), such that subjects who were more likely to use background music responded slower while subjects who found the pleasurable music more familiar responded faster (Figure 9D).

### Helsinki inventory of music and affective behaviors musicianship comparisons

To see how individual listening behaviors varied across different musical backgrounds, we compared the standardized scores (from 0 to 1) of musicians, amateur musicians, and non-musicians on each of the HIMAB variables (except for those directly related to musical experience) with independent samples *t*-tests. For cognitive use of music, non-musicians (mean = 0.40 ± 0.16) scored significantly lower than both musicians [mean = 0.55 ± 0.16; *t*_(49)_ = 3.40, *p* < 0.005] and amateur musicians [mean = 0.53 ± 0.16; *t*_(48)_ = 2.84, *p* < 0.01], indicating less cognitive motivation for listening to music (N-M < AM, M). In terms of music engagement, non-musicians (mean = 0.47 ± 0.05) again scored significantly lower than musicians [mean = 0.61 ± 0.05; *t*_(49)_ = 2.06, *p* < 0.05] and amateur musicians [mean = 0.73 ± 0.23; *t*_(48)_ = 3.77, *p* < 0.0005; N-M < AM, M], consistent with previous findings (Kantor-Martynuska and Fajkowska, in preparation). Finally, musicians (mean = 0.97 ± 0.06) considered music significantly more important in their daily lives than non-musicians did [mean = 0.83 ± 0.18; *t*_(49)_ = 3.45, *p* < 0.005; M > N-M]. No other group differences were significant (all *p*s > 0.06).

## Discussion

Musical pleasure is closely linked to the dopaminergic reward system (Blood et al., 1999; Blood and Zatorre, 2001; Menon and Levitin, 2005; Salimpoor et al., 2011, 2013), but the practical implications of this relationship have not yet been explored. Nonetheless, the rewarding aspects of music are likely due at least in part to musical reward prediction errors (Meyer, 1956; Sloboda, 1991; Huron, 2006; Vuust and Kringelbach, 2010; Salimpoor et al., 2013), and these could have considerable influences on cognitive performance during music listening. We investigated the capacity of subjective musical pleasure to influence reinforcement learning via non-pharmacological dopamine elicitation. Seventy-three subjects of varied musical backgrounds chose pleasurable and neutral musical excerpts from an experimenter-chosen valence-, energy-, and tension-controlled database and reported their musical experiences and listening patterns in the HIMAB (Table 1). In the PS task, they then learned to distinguish between frequently and infrequently rewarded stimuli in three image pairs, and ultimately generalized these relative reward contingencies to recombined pairs of the same stimuli during a test phase (Figure 2). Pseudo-random group assignments determined whether subjects heard pleasurable music or neutral music during the training and test phases of the PS task (Table 1); these group assignments were termed “PN,” “NP,” “PP,” and “NN.” We found that musical pleasure affected task performance in various ways that were consistent with enhanced dopamine transmission, and that these influences depended on the musical backgrounds and listening patterns of the subjects.

### Learning stimulus-outcome relationships

Subjects began the PS task on equal footing even if they had different musical backgrounds or listened to different music. Since accuracy and working memory recruitment as measured by win-stay/lose-switch behavior did not differ across musical experiences or musical conditions, we can assume that the different subject groups were equally naïve to the paradigm and that the effects we observed were due to learning and generalizing during the experiment and not to *a priori* group differences. Notably, while there was already an effect of musical experiences on music-mediated reaction times in the beginning of training, the direction of this effect reversed from training to testing. As such, this cannot be interpreted as an *a priori* bias, but as an immediate influence of the experimental manipulation. Throughout the training phase, the 73 subjects learned to choose the more frequently rewarded images, and their accuracy and reaction times improved from the beginning to the end of training and from the hardest to the easiest training stimulus pair.

Overall, pleasurable music generally accelerated reaction times during learning. These reaction time differences cannot be attributed to arousal because arousal was treated as a covariate and because training reaction times failed to relate to the subjective arousal ratings of the pleasurable music. Moreover, responses were slower for more arousing neutral music (Figures 8B, 9B), and subjects typically considered the pleasurable music more arousing (Table 3). Instead, previous research with reinforcement learning has shown that faster reaction times are associated with greater striatal dopamine efficacy (Caldú et al., 2007; Frank et al., 2007b; Niv et al., 2007; Frank et al., 2009), suggesting that this is a dopaminergic effect. Subjective pleasure can have considerable influences on cognitive performance such that even slight mood changes can alter reinforcement learning, and this effect is thought to rely on enhanced dopamine transmission (Carpenter et al., 2013; for a review see Ashby et al., 1999). Consistent with this, we found that higher subjective pleasantness ratings of the neutral music corresponded to faster reaction times (Figures 8B, 9B), demonstrating that even within the neutral musical condition, responses accelerated when subjects enjoyed the music more.

Musical pleasure also influenced training accuracy. Although this effect was not directly evident when comparing pleasurable and neutral music overall, an interaction between years of musical experience and the music heard during learning revealed that more musically experienced subjects performed more accurately with neutral music and less accurately with pleasurable music (Figure 5).

Together, these results show that musical pleasure enhanced approach behavior during the training phase of the PS task. Importantly, this effect was driven by subjects with little to no musical experience, since subjects with more musical experience instead tended to perform better when listening to neutral music during training and not pleasurable music (Figures 5, 6). These dissociable musical background effects underline the magnitude of the influence that musical pleasure had on subjects with little musical experience, who also reported considering music less important and approaching music less cognitively than other subjects did. Both of these correlations suggest that these subjects devoted less attention to the music than others, and low scores on both of these factors—as well as years spent playing music—were associated with faster training reaction times (Figures 8B, 9B). However, this does not mean that musically inexperienced subjects were unmoved by the music; on the contrary, with less analytical approaches these subjects were probably more emotionally affected (Istók et al., 2009; Müller et al., 2010). In fact, non-musicians rated their pleasurable music higher than musicians did. Thus, these subjects likely devoted fewer cognitive resources to the pleasurable music but enjoyed it more than others, allowing them to attend to learning and simultaneously benefit from the affective, possibly dopaminergic, effects of pleasurable music. Consistent with this interpretation, low cognitive use of music and music importance scores—but also high music engagement and emotional use of music scores—also corresponded to better training accuracy (Figures 8B, 9B), which likely reflects musically inexperienced subjects analyzing the music less but still engaging with it emotionally and enjoying it more. Accordingly, these subjects performed better when they enjoyed the music more (Figure 5).

Reward prediction errors offer a potential mechanism for these findings. With less musical experience and analytical listening than others (Istók et al., 2009), musically inexperienced subjects could be less able to develop reasonable top-down, explicit expectations about the music and thus more susceptible to musically elicited prediction errors (Huron, 2006; Müller et al., 2010; Vuust and Kringelbach, 2010). These greater reward prediction errors would amplify the perceived value of the rewarded stimuli and the music for these subjects (Montague et al., 1996; Schultz, 2002), which would in turn promote approach behaviors (Frank et al., 2004; Caldú et al., 2007; Frank et al., 2007b). Although there are other possible explanations for this result, this interpretation is consistent with recent evidence linking music enjoyment to the reward system and prediction errors (Menon and Levitin, 2005; Salimpoor et al., 2011, 2013).

More musically experienced subjects exhibited opposite reaction time and accuracy patterns. Both musicians and amateur musicians in this study rated music as more important and used music more cognitively than non-musicians, and when considering that musicianship is associated with various advantages in high-level automatic music processing (Koelsch et al., 1999; Tervaniemi et al., 2005; Oechslin et al., 2012) as well as more analytical listening strategies (Istók et al., 2009; Müller et al., 2010), we can infer that they probably devoted more cognitive resources to the music during the PS task. Musicians in this study also gave lower pleasantness ratings for their pleasurable music than non-musicians did, demonstrating that they did not enjoy the musical stimuli (which were clips from film soundtracks) as much as other subjects did, even though they also reported engaging with music more. Interpreting these results from the perspective of more musical experience and more analytical listening, musicians can be said to have more “critical ears” than other listeners. More musically experienced subjects might have therefore been less emotionally affected by their pleasurable music, chosen as it was from a limited amount of pieces predetermined by the experimenters. Nonetheless, with more cognitive listening strategies, these subjects might have been more inclined to analyze music the music they preferred, even if the margin of preference was minimal. This could explain why more musically experienced subjects performed better with neutral music than with pleasurable music (Figures 5, 6), and why cognitive use of music and music importance were both more prevalent among musicians and simultaneously related to decreased training accuracy (Figures 8A, 9A).

### Generalizing about probabilistic rewards

By the end of training, the 73 subjects included in the analysis had learned to choose the more frequently rewarded stimuli. They thus entered the test phase with sufficient task knowledge, albeit with differences demonstrating that the musical manipulation was already influencing task performance. After 54 presentations of each training pair, subjects transferred what they had learned to a test phase with no feedback. The test included all possible combinations of the six training images: the three training pairs plus 12 novel combinations.

Despite responding faster with neutral music in the training phase, more musically experienced subjects responded to test stimuli faster when they listened to pleasurable music. Specifically, musically experienced subjects who listened to neutral music during training (and thus responded faster in that phase) exhibited slower reaction times during the test phase if they then listened to neutral music and quicker reaction times if they then listened to pleasurable music. The less musically experienced subjects, alternatively, responded to neutral music with slower reaction times during training but then faster reaction times during testing (Figure 7). A trend effect of musical condition suggesting faster reactions during pleasurable music implied that more musically experienced subjects drove this effect this time in spite of a sample that was skewed toward the less experienced.

As discussed above, the HIMAB results suggest more musically experienced subjects were more likely to focus on the music they enjoyed during the task. This was detrimental to their training performance during pleasurable music listening, but the same behavior could have had the opposite effect during the test. While learning about relative reward contingencies involves predictions, prediction errors, valuation, salience attribution, and working memory processes (Schultz, 2002; Jocham et al., 2011; Collins and Frank, 2013), performance on a test without feedback depends more on motivation and the management of previously learned values (Robinson and Berridge, 1993; Jocham et al., 2011; Shiner et al., 2012). In other words, expressing reinforced behaviors is considerably less cognitive than acquiring them (Doll et al., 2011). As such, devoting cognitive resources to music would not detract from performance on the mostly non-cognitive test in the same way that it detracted from training performance. This could explain why the test music had a greater influence on musically experienced subjects than the training music, and why the beneficial effect of pleasurable music on musically inexperienced subjects during training seemed to disappear when these subjects transferred their task knowledge to the test phase. With musically experienced subjects less cognitively engaged in the PS task during testing and thus suddenly more susceptible to the musical background, the behaviors of less musically experienced subjects were likely overshadowed by this dramatic shift. The contrasting results for more and less musically experienced subjects, then, could once again reflect their more and less cognitive listening strategies, respectively.

In many ways, the behaviors of experienced music listeners in this study resembled those of experienced musicians. Results for music playing years and weekly music listening hours paralleled each other throughout both training and testing, even though the former measured music-making and the latter only music listening. Since this experiment involved listening to but not making, reading, or writing music, subjects who regularly listened to a lot of music behaved similarly to those with extensive musical training when they performed a task with a musical background (cf. Bigand and Poulin-Charronnat, 2006). This can also be seen in terms of individual music consumption, for which higher scores corresponded to better training accuracy (Figures 8A, 9A) and faster reaction times during training (Figures 8B, 9B) and testing (Figure 8D).

Although several of the present findings are consistent with enhanced approach behavior during pleasurable music listening, we observed a “NoGo” bias in our data throughout the test. In addition, subjects who never listened to pleasurable music during the PS task (the NN group) performed the worst at avoiding the most frequently punished stimulus, while those who listened to pleasurable music once (NP and PN) performed the best (Figure 4). Although we expected to find an approach bias due to pleasurable music, this finding is not unprecedented in healthy subjects (Jocham et al., 2011). The presence of music might have distracted subjects from the task at hand to the extent that it was actually somewhat aversive, which could account for the avoidance bias we observed. At the same time, the pleasurable music condition would have been less aversive than the neutral music condition, and this can explain the relative approach effects we found with pleasurable music compared to neutral music.

Alternatively, subjects distracted by music could have been less reliable than normal in their choices of A over B during learning, which would result in more than the typical amount of punishments after choosing B and thus lead to more of an avoidance bias than usual. As such, our data could exhibit an overall avoidance bias due to music in general, with the differential group effects merely reflecting the overall group effects that only reached significance in the test trials for which the avoidance-biased subjects were especially prepared. Again assuming that subjects attended more to music they preferred, this would imply that they were more distracted by pleasurable music than by neutral music, and thus more likely to receive negative feedback when learning with pleasurable music. Listening to pleasurable music for the second time in a row, however, would not have been equally engaging. Even so, the NP group was best at avoiding B, which could simply reflect the aforementioned advantage enjoyed by musically experienced subjects in this group. Indeed, this effect represents a subset of the test phase, during which the behavioral shift in musically experienced subjects (especially in the NP group) had a profound influence.

### Individual factors

We assessed the relationships between performance in the PS paradigm and musical experiences, different uses of music (Chamorro-Premuzic and Furnham, 2007), music consumption (Chamorro-Premuzic et al., 2012), music-directed attention (Kantor-Martynuska and Fajkowska, in preparation), music importance, active and passive listening frequencies, and subjective ratings of the pleasurable and neutral music in the study.

These factors greatly shaped learning, with musical experience, uses of music, music consumption, music-directed attention, music importance, listening frequencies, and subjective ratings from the listening test all influencing training accuracy or reaction times (Figures 8A,B, 9A,B) and background use of music, music consumption, music importance, active listening, and subjective ratings of the experimental music affecting test performance (Figures 8C,D 9C,D). As discussed above, higher music importance and cognitive use of music scores were associated with both worse training accuracy and slower training reaction times, more emotional music listening corresponded to better training accuracy, more years of playing music were associated with faster training reaction times, more music engagement was correlated with better training accuracy, and greater music engagement scores corresponded to faster training responses. Notably, subjects who devoted more time to active music listening without any distractions tended to respond less accurately but more rapidly during training, suggesting that they probably tried to listen to the music actively during the PS task and devoted fewer cognitive resources to the PS task, perhaps responding impulsively due to lack of focus. Subjects who spent more time passively listening to music as one of many tasks also tended to be less accurate during learning, and those who used music for background purposes more were both less accurate and slower to respond. One possible interpretation for these counter-intuitive findings is that individuals who normally listen to music while doing non-cognitive tasks might have been distracted by the cognitive nature of the PS task's training phase. Another explanation could be that subjects who were more likely to listen to music passively and in the background, as opposed to actively and in the foreground, are also less likely to become invested in music and respond to it emotionally, using it instead simply to fill what would otherwise be silence. Neither of these interpretations conflicts with the finding that subjects with greater music consumption scores were both more accurate and quicker to respond, most likely due to their greater exposure to music. Finally, music distractibility, which measures the extent to which music diverts attention from a primary focus such as the PS task, also corresponded to decreased training accuracy and slower training reaction times.

Subjective ratings of the music played during the PS task also correlated to task performance. As discussed earlier, higher subjective pleasantness ratings of the neutral music correlated to faster reaction times during training, probably because the neutral music condition for these subjects was not as aversive as it was for others. Likewise, these ratings also increased with greater training accuracy. Higher arousal ratings of the neutral music were correlated with decreased accuracy and slower reaction times, whereas higher arousal ratings of the pleasurable music correlated to greater accuracy. Once again, the higher ratings within each musical condition could have exaggerated the aversive and pleasurable effects of that condition, respectively. Greater familiarity ratings of the pleasurable music quickened training reaction times, possibly because this more predictable music was less distracting, and/or because more familiar pleasurable music is likely to be more pleasurable than unfamiliar pleasurable music as evidenced by behavioral and fMRI findings (Pereira et al., 2011). Altogether, these subjective differences altered task performance according to the affective experience of the listener, but as discussed above that experience depended largely on musical background.

During the test, several HIMAB results mirrored those of training: greater music consumption correlated to faster responses, more active listening again corresponded to faster reaction times, greater music importance was associated with slower reaction times, and higher background use of music scores related to slower responses and worse accuracy, just as in the training phase. This last result, regarding background use of music, is consistent with the aforementioned interpretation that subjects who listen to background music are less likely to respond to it emotionally. However, this finding is not consistent with the interpretation that the cognitive nature of the PS task distracted these subjects, since the test phase of this task is considerably less cognitive than the training phase (Doll et al., 2011). Consequently, it seems that subjects more likely to use music for background purposes were less likely to become emotionally invested in the music during the PS task.

Also as in training, higher familiarity ratings of the pleasurable music corresponded to faster test responses. Likewise, higher familiarity ratings of the neutral music were associated with slower test responses as well as greater accuracy, possibly because more familiar neutral music was more enjoyable to some but more tedious to others. Finally, higher pleasantness ratings of the neutral music were correlated with greater test accuracy, consistent with the same result during training.

Overall, these results imply that learning strategies differ greatly across individuals (cf. Fabry and Giesler, 2012) and depend on several factors, whereas generalizing about previously learned information depends more on the context of the test than on background factors. This finding is consistent with our observations of different listening strategies between more and less musically experienced subjects that had greater influence in the more cognitive learning phase of task at hand. Put another way, each individual's approach to learning depended largely on his or her musical background, but expressing previously learned knowledge was a less cognitive task that thus allowed for more of an immediate emotional effect in even the more analytical music listeners.

### Limitations and conclusion

The present study represents a first step in bringing together musical pleasure and reinforcement learning to explore their common roots in the cognitive neuroscience of reward. Using a reinforcement learning task to study the rewarding aspects of music listening, we found that pleasurable music was able to influence task performance in the expected way: that is, in a way consistent with the actions of a dopamine agonist. Examining inter-individual differences, we revealed complex effects of musical pleasure on reinforcement learning that depended on the musical backgrounds and listening behaviors of the subjects.

Listening to pleasurable music activates areas of brain implicated in emotion and reward (Blood et al., 1999; Blood and Zatorre, 2001; Menon and Levitin, 2005; Salimpoor et al., 2011, 2013). Our findings suggest that musical pleasure acted on the dopaminergic reward system because it influenced performance in a task dependent on dopamine transmission (Frank et al., 2004), but we did not directly measure dopamine transmission in any way. Other neurotransmitters and systems were likely involved, and the mesocorticolimbic effects of musical pleasure may in fact be insufficient to influence reinforcement learning. Instead, music could alter task performance via attentional, working memory, or sensorimotor influences. In addition to direct measurements of dopamine transmission, neuroimaging the temporal and spatial dynamics of musical pleasure and reinforcement learning would elucidate their interactions as well as the various contributions of brain areas involved in attention, memory, and motion. Future research would also benefit from direct measures of attention, working memory, and sensorimotor integration during music listening and/or task performance, as well as music listening information that reflects the subjects' real-time behaviors during the experimental task. Objective physiological measures of pleasure and arousal, shown to correlate to one another (Salimpoor et al., 2009), would also be an improvement on the subjective ratings we used in the present study. Finally, the learning effects we observed could reflect group differences in intelligence or learning aptitude, which could be controlled for in subsequent investigations.

Selecting stimuli for neuroaesthetics research is necessarily problematic. When experimenters choose, participants are prone to disagree with their judgments and enjoyment will vary across individuals. When participants choose, stimuli are likely to differ tremendously and skew the sample (e.g., toward faster, happier music instead of a balanced range). Salimpoor and colleagues (2009, 2011) introduced a method wherein each participant's favorite music served as another participant's neutral music, and we adapted this technique by pre-selecting 14 instrumental pieces of similar valence, energy, and tension from which each subject could choose. We ensured that subjects enjoyed their pleasurable music but not their neutral music, and we used familiarity, pleasantness, and arousal ratings as covariates in our analyses. However, even this combination of experimenter-selected and participant-selected methods limits the range of enjoyment our subjects felt in exchange for a more controlled stimulus set.

Most experiments that investigate the differences between learning and testing ignore training response times (Jocham et al., 2011; Shiner et al., 2012). In the present study, musical pleasure differentially influenced reaction times according to musical experience during learning, which would not have been apparent by analyzing accuracy alone. Moreover, these effects were shaped by several factors that varied across individuals. Subjectivity seems to have profound effects on dopamine transmission, implying that the discordant conclusions of previous reinforcement learning research could arise from complex interactions between innate predispositions, neuroplastic changes, and experimental manipulations underlying dopamine efficacy. Though there is a growing body of research on the ability of dopaminergic agonists/antagonists to influence instrumental learning in neuropsychiatric disorders (e.g., Frank et al., 2004; Chase et al., 2010; Worbe et al., 2011; Grob et al., 2012), no study that we know of has investigated the relationships between individual factors and dopaminergic manipulations in healthy subjects. We found subjective modulations of music's effects, signifying that our enjoyment of music depends a great deal on the amount of music we listen to, how we listen to it, how we engage with it, our musical experience, and even our reasons for approaching it. Since this is the first study we know of to apply individual musical background and listening factors to background music listening during an experimental task, our interpretations of these results represent only a subset of the possible explanations for these effects. Future research should further investigate the influences of different individual experience and listening behaviors on musical and non-musical tasks, as well as their mechanisms. Nonetheless, these factors all seem to influence the rewarding impact of music, signaling the need for a more subjectivist approach to musical pleasure and reward.

Music is a powerful and universal phenomenon, intensely important and rewarding to many people (Sloboda and Juslin, 2001; Dubé and Le Bel, 2003). Musical pleasure thus offers an ecological and dynamic approach to investigating reward, while reward itself offers many practical applications for musical pleasure. Bringing these topics together, then, has important implications in education, affect, and therapy. In particular, Parkinson's disease represents a promising avenue for future research since the relationship between this disease and reinforcement learning is very well understood (Frank et al., 2004, 2007b; Shiner et al., 2012) and music therapy has already been shown to improve motor and cognitive deficits in Parkinson's disease (Pacchetti et al., 2000). Ultimately, whether or not our findings reflect altered dopamine transmission remains to be seen, but the ability of musical pleasure to influence reward-based decision making speaks to its affective and effective potency.

### Conflict of interest statement

The authors declare that the research was conducted in the absence of any commercial or financial relationships that could be construed as a potential conflict of interest.

## Appendix

**Table A1:** **Musical stimuli**.

**Excerpt**	**Soundtrack**	**Track**	**Title**	**Artist**	**Length**
1	Pride and prejudice				4:49 (Total)
		9	Liz on top of the world	Jean-Yves Thibaudet	1:24
		13	Darcy's letter	Various	3:26
2	Pride and prejudice				7:20 (Total)
		3	The living sculptures of pemberley	Various	3:04
		15	Your hands are cold	Jean-Yves Thibaudet	4:21
3	Juha				3:08 (Total)
		16	Kevät	Anssi Tikanmäki	1:01
					
		19	Rakkauden Uhrit	Anssi Tikanmäki	2:41
4	Lethal weapon 3	10	Lorna—a quiet evening by the fire	Michael Kamen/Eric Clapton	3:33 (Total)
					
5	Shakespeare in love				7:54 (Total)
		2	Viola's Audition	Nick ingman/Gavyn wright	3:22
		3	A plague on both your houses	Nick ingman/Gavyn wright	1:40
		6	In Viola's room	Nick ingman/Gavyn Wright	2:54
6	Dances with wolves				4:15 (Total)
		2	The John Dunbar theme	John Barry	2:17
		4	Ride to fort hays	John Barry	2:01
					
7	Big fish				5:39 (Total)
		8	Pictures	Danny Elfman	0:45
		11	Underwater	Danny Elfman	1:53
		18	In the Tub	Danny Elfman	1:18
		22	Jenny's Theme	Danny Elfman	1:45
8	Shine				3:49 (Total)
		18	As if there was no tomorrow	David Helfgott	1:46
		28	Goodnight daddy	David Helfgott	2:05
9	Pride and prejudice				4:31 (Total)
		1	Dawn	Various	2:40
		12	The secret life of daydreams	Jean-Yves Thibaudet	1:56
10	Portrait of a lady				7:04 (Total)
		3	Flowers of Firenze	Wojciech Kilar	4:02
		4	Twilight Cellos	Wojciech Kilar	3:07
11	Oliver twist	2	The road to the workhouse	Rachel Portman	3:03 (Total)
12	The last samurai	1	A way of life	Hans Zimmer	8:04 (Total)
13	Dances with wolves				5:56 (Total)
		8	Kicking bird's gift	John Barry	2:11
		12	The love theme	John Barry	3:46
14	Band of brothers				6:22 (Total)
		12	Headscarf	Michael Kamen	4:12
					
		15	Preparing for patrol	Michael Kamen	2:13

**Table A2:** **Helsinki inventory of music and affective behaviors**.

**A. MUSICAL TRAINING**
1a. Have you learned to play an instrument or been in a choir? (Yes or No)
If you answered “No,” please continue to the “Listening to music” section.
1b. How many years have you taken instrumental or singing lessons?
1c. How old were you when you started learning an instrument (including voice)?
1d. If you learned to play an instrument (including voice) and then stopped, how old were you when you stopped?
2a. Are you or were you a professional musician or music student? (Yes or No)
If you answered “No,” please continue to question #3.
2b. What was your main instrument? Did you play other instruments?
2c. How many years have you played/did you play music professionally or as a student?
3. Currently, how much time per week do you practice or play one or more instruments or sing?
4. Which of the following describes you the best? (Write in one or more musical styles that best describes your musicianship).
Pop/jazz/heavy/folk/classical/________ musician
Pop/jazz/heavy/folk/classical/________ musical enthusiast/amateur
Write your genre(s) here:
**B. LISTENING TO MUSIC**
1. How often do you actively listen to music (without doing something else at the same time)?
Never
Once per year
Once per month
2–3 times per month
Once per week
2–3 times per week
More often (How many hours per week?)
2. How often do you listen to music passively (e.g., while you are cleaning, etc.)?
Never
Once per year
Once per month
2–3 times per month
Once per week
2–3 times per week
More often (How many hours per week?)
3. Please evaluate how important music is in your daily life
Not at all important 1 ——— 2 ——– 3 ——– 4 ——— 5 ——– 6 ——— 7 Very important
**C. MUSIC CONSUMPTION (ADAPTED FROM Chamorro-Premuzic et al., 2012)**
Using the scale below, please indicate how frequently you engage in each of the following activities.
Very rarely 1 ——— 2 ——– 3 ——– 4 ——— 5 ——– 6 ——— 7 Very often
I purchase or download music…
I attend musical concerts or recitals…
**D. USES OF MUSIC (FROM Chamorro-Premuzic and Furnham, 2007)**
Using the scale below, please indicate the extent to which you agree or disagree with each of the following activities. Please write a number after each activity.
Strongly disagree 1 ——— 2 ——– 3 ——– 4 ——— 5 ——– 6 ——— 7 Strongly agree
1. Listening to music really affects my mood.
2. I am not very nostalgic when I listen to old songs I used to listen to.
3. Whenever I want to feel happy I listen to a happy song.
4. When I listen to sad songs I feel very emotional.
5. Almost every memory I have is associated with a particular song.
6. I often enjoy analyzing complex musical compositions.
7. I seldom like a song unless I admire the technique of the musicians.
8. I don't enjoy listening to pop music because it's very primitive.
9. Rather than relaxing, when I listen to music I like to concentrate on it.
10. Listening to music is an intellectual experience for me.
11. I enjoy listening to music while I work.
12. Music is very distracting so whenever I study I need to have silence.
13. If I don't listen to music while I'm doing something, I often get bored.
14. I enjoy listening to music in social events.
15. I often feel very lonely if I don't listen to music.
**E. MUSIC-DIRECTED ATTENTION SCALE (FROM Kantor-Martynuska and Fajkowska, in preparation)**
These questions regard listening to music at a medium volume. For each sentence, choose the answer that is more relevant to your experience. Respond quickly, according to the first decision that comes to your mind. (Agree or Disagree)
1. When I eat out, music playing in the background is of no importance to me.
2. I turn off my music and go out only after the piece of music I'm listening to has finished.
3. When I have a difficult mathematics task to do, music disturbs me.
4. Background music diverts my attention from what another person is saying to me.
5. I don't mind if I have to stop a piece of music halfway through.
6. When I eat, inappropriate music disturbs me.
7. When I hear someone else's music playing through his/her earphones, I can detach myself from the music if I want.
8. When I have to write an essay, I do it with the music on.
9. Even when I am concentrating on something, I like to have the music on.
10. In a conversation, I can be distracted by music playing in the background.
11. When I study for an exam, music playing in another room distracts me.
12. When I hear music, I find it hard not to listen to it attentively.
13. I am more effective when I study in silence than with the music on.
